# Improved GBS-YOLOv5 algorithm based on YOLOv5 applied to UAV intelligent traffic

**DOI:** 10.1038/s41598-023-36781-2

**Published:** 2023-06-13

**Authors:** Haiying Liu, Xuehu Duan, Haitong Lou, Jason Gu, Haonan Chen, Lingyun Bi

**Affiliations:** 1 The School of Information and Automation Engineering, Qilu University of Technology (Shandong Academy of Sciences), Shandong, China; 2 The School of Electrical and Computer Engineering, Dalhousie University, Halifax, Canada

**Keywords:** Engineering, Electrical and electronic engineering

## Abstract

As the road traffic situation becomes complex, the task of traffic management takes on an increasingly heavy load. The air-to-ground traffic administration network of drones has become an important tool to promote the high quality of traffic police work in many places. Drones can be used instead of a large number of human beings to perform daily tasks, as: traffic offense detection, daily crowd detection, etc. Drones are aerial operations and shoot small targets. So the detection accuracy of drones is less. To address the problem of low accuracy of Unmanned Aerial Vehicles (UAVs) in detecting small targets, we designed a more suitable algorithm for UAV detection and called GBS-YOLOv5. It was an improvement on the original YOLOv5 model. Firstly, in the default model, there was a problem of serious loss of small target information and insufficient utilization of shallow feature information as the depth of the feature extraction network deepened. We designed the efficient spatio-temporal interaction module to replace the residual network structure in the original network. The role of this module was to increase the network depth for feature extraction. Then, we added the spatial pyramid convolution module on top of YOLOv5. Its function was to mine small target information and act as a detection head for small size targets. Finally, to better preserve the detailed information of small targets in the shallow features, we proposed the shallow bottleneck. And the introduction of recursive gated convolution in the feature fusion section enabled better interaction of higher-order spatial semantic information. The GBS-YOLOv5 algorithm conducted experiments showing that the value of mAP@0.5 was 35.3$\%$ and the mAP@0.5:0.95 was 20.0$\%$. Compared to the default YOLOv5 algorithm was boosted by 4.0$\%$ and 3.5$\%$, respectively.

## Introduction

Since the 20th century, object detection has become an important research result in the field of computer vision. It has been widely developed and applied in the fields of intelligent transportation, pedestrian detection and driverless driving^1–4^. As the accuracy of image classification improves, object detection algorithms based on convolutional neural networks^5,6^ have gradually become an current research hotspot.

UAV technology^7^ has become an important application area for drones with the in-depth study of relevant theory and practice. With the creation of high-precision sensors and the development of related technologies, the performance of UAVs is constantly being improved. It is also widely used in pedestrian detection, daily patrols and vehicle detection^8^. Drones are often equipped with high-definition cameras for image acquisition, with the advantages of easy operation, flexible flight, good stability, low cost, etc. In recent years, with the development of computer vision technology, the application of UAV intelligent transportation based on computer vision detection technology has received more and more attention from domestic and foreign research scholars.

For urban traffic management, both population and vehicle detection is an important detection object. Dense crowds and mixed traffic with non-motorized vehicles are the main factors causing traffic congestion and gridlock^9^. In addition, each city has a limited number of traffic police officers, and when there are traffic jams or situations requiring police patrols in many areas of the city, a large number of police officers need to be mobilized to maintain social order. This is not only labor demanding, but also less work efficient. The utilization of drones for daily crowd detection in priority places as well as vehicle detection^10^ on key roads not only brings efficiency improvements to the daily work of traffic police, but also offers a higher level of security.

Most current object detection algorithms are generally aimed at normal sized objects. And these algorithms perform well on most open source datasets^11^, such as the Pascal VOC dataset (VOC)^12^, Common Objects In Context dataset (COCO)^13^, and the ImageNet dataset^14^. The COCO dataset, common to the target detection field, contains more than 330,000 images and 80 categories^15^. It is also classified into large-size target^16^ (objects with area larger than 96 $\times$ 96), medium-size target^16^ (objects with area between 32 $\times$ 32 and 96 $\times$ 96), and small-size target^16^ (objects with area smaller than 32 $\times$ 32). The datasets such as COCO and VOC include medium and large size targets occupying most of them. The performance of target detection models in datasets such as COCO cannot meet the requirements of small target detection. In regard to the above problems, small target dataset^17^ applications for UAV photography were born, such as VisDrone 2019^18^, UAV 123^19^, etc. Compared to the default algorithm, our proposed GBS-YOLOv5 algorithm encountered the following challenges for experiments on the VisDrone dataset^20^:

- There are many targets. Hundreds of targets to be detected in each image, and many targets overlap^21^;
- Target deformation. Different from the conventional^21^ dataset, the shooting angle of the UAV causes the target deformation;
- The size of the target is too small^21^, because it was taken by a drone in the air, far away from the target. The small size of the target in the picture makes normal model training ineffective;
- The background and scene are complex. The dataset contains two kinds of scenes, day and night. The UAV has a large perspective^21^, and the UAV^22^ takes pictures of more complex backgrounds.

**Figure 1:**
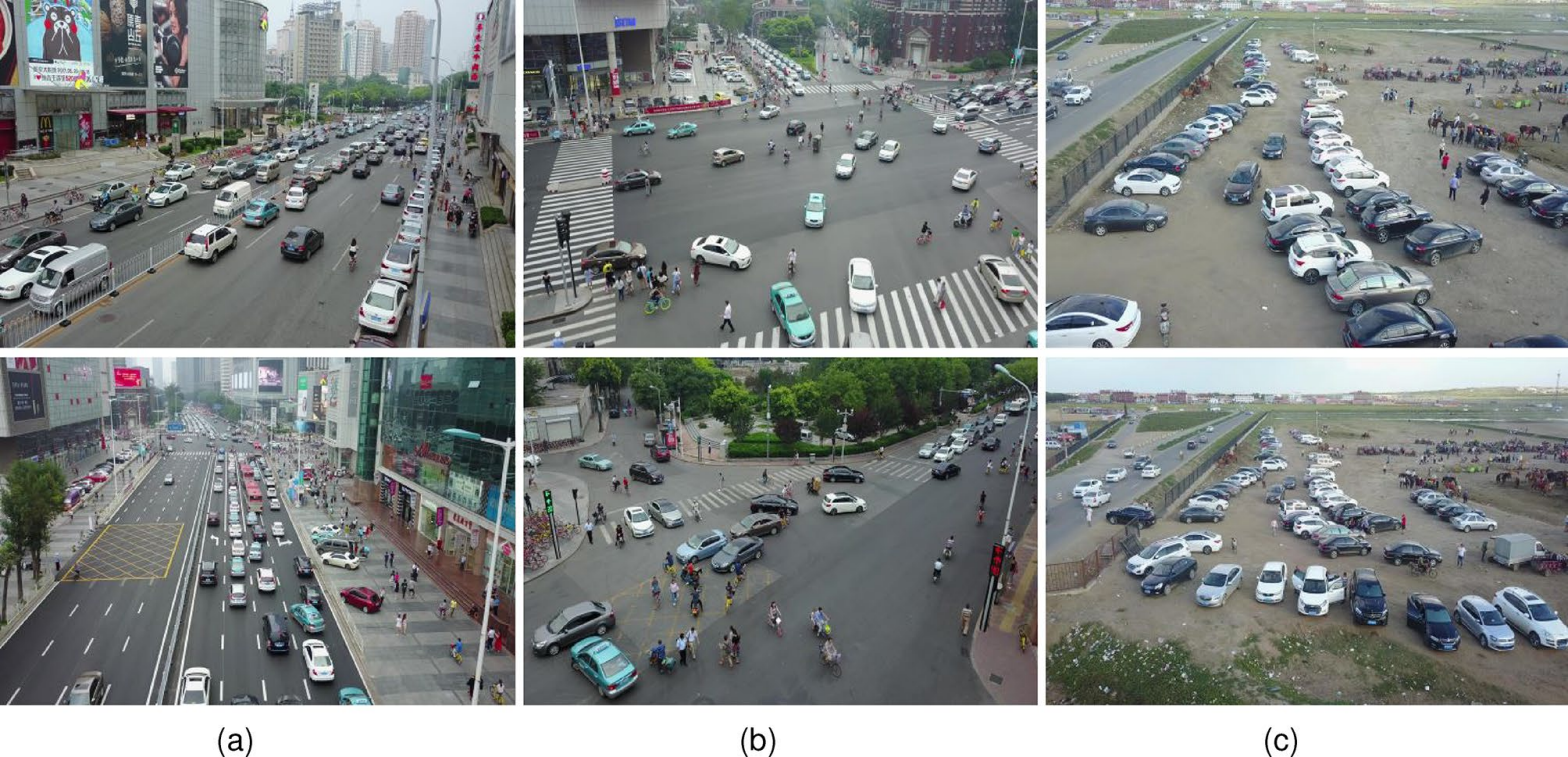
Datasets examples in (**a**) busy street block, (**b**) crossroads, and (**c**) the suburbs.

Some samples of the VisDrone 2019 dataset are shown in Fig. 1.

The improved algorithm was mainly based on the default CSP-Darknet53 (Cross Stage Partial Darknet53)^23^. Introduced efficient spatio-temporal interaction (ESI) module, and the C3 (Cross Stage Partial Bottleneck with 3 convolutions) module^24^ was replaced in Backbone^24^ with ESI module. The shallow bottleneck (SB) module was used to retain more shallow semantic information^25^, thus solved the problem of large loss of small target information^26^. We added a fourth detector head to the original network for the output of minimal targets. The spatial pyramid convolution (SPC) module was used to extract the shallow feature information as the fourth detection head. Finally, we introduced PAN (Path Aggregation Network) feature fusion^27^ into recursive gated convolution^28^ in the Neck part of YOLOv5^29^. This could better realize the semantic information interaction of high-order spatial features^30^. Through experiments, the test results based on the VisDrone dataset showed that GBS-YOLOv5 was 4.0$\%$ and 3.5$\%$ higher than the original YOLOv5 algorithm under the conditions of IoU of 0.5 and 0.95, respectively.

To sum up, our main contributions are as follows:

- An efficient spatio-temporal interaction module was proposed. It could better preserve spatial feature information, deepen the depth of feature extraction^31^ network, and mine more abundant feature information^32^ of the target;
- The shallow bottleneck module was added to the Backbone structure to enrich the shallow feature layer information;
- Added SPC detection head. Used the pyramidal convolution module as a feature extraction^33^ module for small targets;
- Recursive gated convolution was combined to the Neck part^34^ of feature fusion to realize the semantic information interaction of high-value spatial features.

## Realted work

With the rapid development of artificial intelligence technology^35^ since the 20th century. Target detection algorithm based on depth learning^36^ has become the mainstream target detection algorithm with its advantages of simple structure and higher accuracy. There are mainly two research directions: one-stage^37^ network model and two-stage^38^ network model.

The two-stage model divides the target detection problem into two stages: extracting candidate regions^39^, classifying candidate regions and position correction^39^. Object detection is realized through these two stages.In 1998, Yann Le Cun et al. first proposed the application of convolutional neural network to digital recognition^40^. In 2012, Alex Krizhevsky and others proposed the AlexNet algorithm and won the championship in the ImageNet 2012 Challenge^41^ Since then, target detection based on deep learning has entered a stage of rapid development. In 2015, Girshick R realized the shared convolution operation based on the R-CNN (Regions with CNN features)^42^ algorithm and proposed Fast R-CNN^43^. Later, Faster R-CNN^44^ was proposed. He Kaiming put forward Mask R-CNN^45^ in 2017, realizing the task of segmentation^46^.

**Figure 2:**
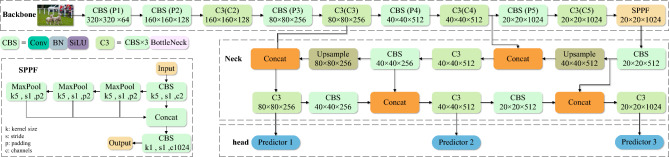
The default YOLOv5 network structure.

Due to the two-stage is based on the classification of the target detection algorithm, the detection speed is slow^47^, and still can not meet most of the requirements of real-time places. Therefore, one stage target detection algorithm is proposed to solve the real-time problem^48^ of detection. Among those, the one stage detection algorithms mainly include mainstream algorithms such as YOLO (You only look once)^49^, SSD (Single Shot MultiBox Detector)^50^ and RetinaNet^51^. In 2016, Redmon J et al. proposed the YOLO algorithm^52^. Unlike the two stages, the algorithm uses the target detection framework as a regression problem^52^. Subsequently, YOLOv2^53^, YOLOv3^54^, YOLOv4^55^, YOLOv5^56^ and YOLOv7^57^ of YOLO series were put forward successively. This series of algorithms are easier to be embedded in mobile devices because of their small amount of computation and fast computing speed. In 2022, F-CenterNet was proposed, which was a UAV target detection algorithm based on a single-stage detection framework. The algorithm used embedded reinforcement learning to adaptively optimize the network structure and loss function. In 2020, Scaled-YOLOv5 was proposed, a lightweight UAV target detection algorithm that used technologies such as multi-resolution training, data augmentation, and adaptive convolution to achieve fast inference while maintaining high accuracy. DroneNet was an integrated framework for UAV target detection and tracking problems, including classic models such as Faster R-CNN, YOLOv2 and had achieved good results by training ensemble models. Our improved algorithm and experimental work are based on YOLOv5 algorithm. The default YOLOv5 network structure is shown in Fig. 2. As can be seen, YOLOv5 is divided into Backbone, Neck and Head sections in total.

The Backbone section is mainly composed of the downsampling module (CBS), the residual module (C3) and the SPPF (Spatial Pyramid Pooling-Fast)^58^. One of these is the CBS module^59^ which is a standard convolution module. It is made up of the convolution operation, the batch norm normalisation process and the SiLU activation function^60^. The C3 module is the main module for learning on residual features. Its structure is divided into two branches. One of the branches uses multiple Bottleneck stacks and three standard convolutional layers. The other one only goes through a basic convolution module. Finally, the two branches are subjected to the concat operation. When the picture is sent into the YOLOv5 detection frame. First, the Backbone convolution module is used to perform the down sampling operation of the feature map. Then, learn the residual characteristics through the residual network and deepen the depth of the network. Next, repeat the above operations and finally pass through the SPPF (Spatial Pyramid Pooling Fast) module. Finally, the feature information is transferred to the Neck part for feature fusion.

The Neck section is made up of FPN^29^ (Feature Pyramid Networks, FPN) and PAN^29^ (Path Aggregation Network, PAN) structures. Where the FPN is a top-down feature fusion from the bottom of the Backbone network to build a high-level semantic feature map. PAN structure carries out feature fusion from bottom to top to make up for strengthened positioning information.

The Head section is made up of predictor 1, predictor 2 and predictor 3. The images undergo feature extraction, feature fusion to eventually form three sizes of detection heads, which are used to output three different sizes of targets: large, medium and small.

In the field of target detection, the performance of target detection algorithms requires certain evaluation criteria to measure how well the algorithm performs. The mAP (mean Average Precision) is a very important measure. In addition, common metrics used to measure target detection algorithms are accuracy^27^, precision^27^, recall^27^, IoU^27^, etc.

IoU: Positioning accuracy can be determined by the window overlap between the detection window and the marked object, i.e. Intersection-Over-Union (IoU). Let the marker window be A and the detection window be B. The IoU is calculated as in Eq. (1).

1 $$\begin{aligned} IoU=\frac{A\bigcap B}{A\bigcup B}. \end{aligned}$$

The numerator represents the area of the overlap between windows A and B, and the denominator represents the sum of the areas of windows A and B. Obviously, the value of IoU is between [0, 1], while the closer the IoU is to 1, the more the two windows overlap and the better the positioning accuracy, and vice versa.

P: represents the precision^49^ rate. It is the ratio of the actual number of positive samples in the predicted sample to the number of all positive samples. The larger the value the better, 1 being ideal. Calculated as in Eq. (2).

2 $$\begin{aligned} Precision=\frac{True\text { }positives}{True\text { }positives+False\text { }positives} \end{aligned}$$

R: represents the recall^49^ rate. This is the ratio of the actual number of positive samples in the predicted sample to the number of samples predicted. Generally speaking, the higher the recall rate, the lower the accuracy rate tends to be. Calculated as in Eq. (3).

3 $$\begin{aligned} Recall=\frac{True\text { }positives}{True\text { }positives+False\text { }negatives} \end{aligned}$$

Map: Mean Average Precision^49^. It averages the accuracy values for multiple validation sets of individuals. As a measure of detection accuracy in object detection. There are two metrics in total. At IoU of 0.5, this is expressed as mAP@0.5. At IoUs of 0.5–0.95, the average mAP on a step size of 0.05 is expressed as mAP@0.5:0.95.

## Improved methods

**Figure 3:**
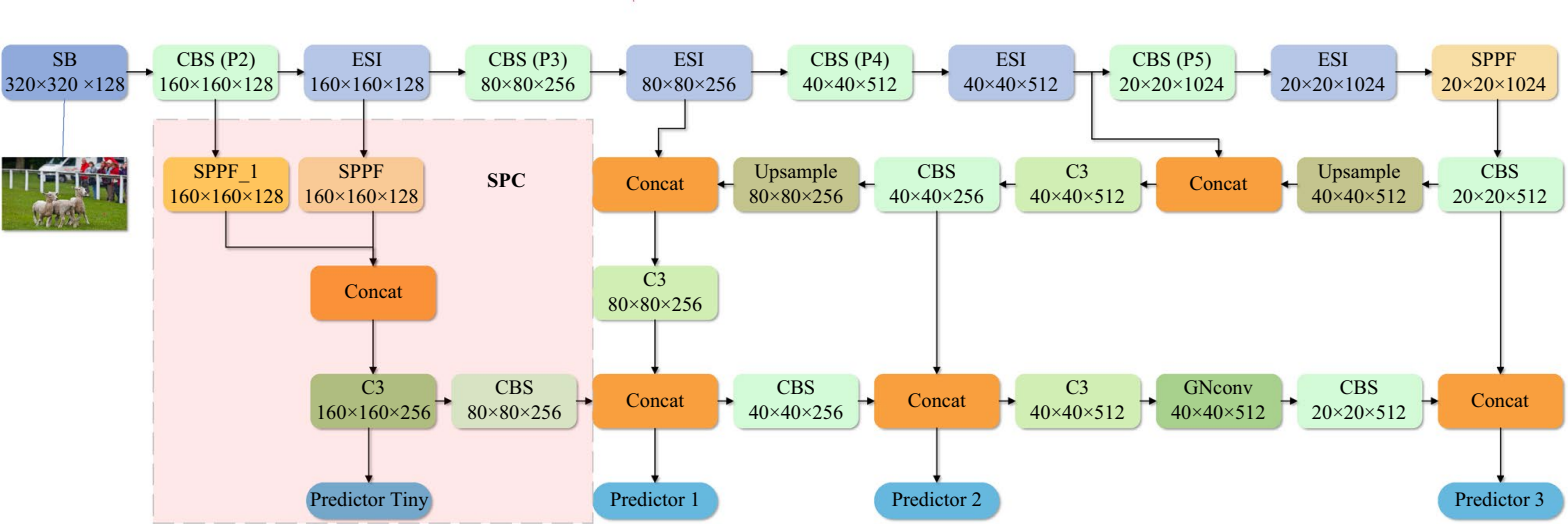
The network of GBS-YOLOv5.

The YOLO series was a deep learning-based target detection framework that achieved efficient target detection by detecting all objects in a single forward propagation. YOLOv7 and YOLOv5 were different versions of YOLO, with YOLOv7 being the newer version. In terms of computational efficiency and accuracy, YOLOv7 improved relative to YOLOv5. However, YOLOv5 was much faster than YOLOv7 in terms of training and inference and had a lower memory footprint. This made YOLOv5 more advantageous in UAV detection application scenarios. Therefore, it remained our choice to improve YOLOv5 version. The network structure of our proposed GBS-YOLOv5 algorithm was shown in Fig. 3. Firstly, at the input of the network, we used a shallow bottleneck to downsample the picture while increasing the number of channels of the feature map. By this it would be possible to retain as much detailed information about the target as possible. Next, our proposed efficient spatiotemporal interaction module was used as the residual feature learning module for Backbone. It could better combine deep semantic information with shallow semantic information. In the Neck section, we added a spatial pyramidal convolution module to the 160 $\times$ 160 layers as the detection head for very small targets. Finally, recursive gated convolution was introduced into the PAN structure to enhance spatial localization information.

### Spatial pyramid convolution module

**Figure 4:**
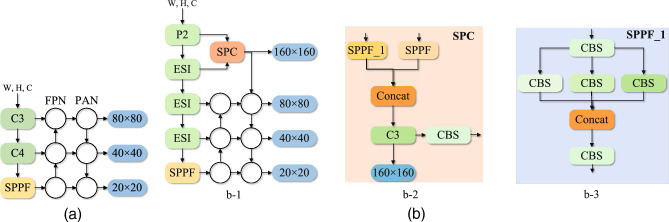
The spatial pyramid convolution module (**a**) default YOLOv5 structure, (**b**) b-1: GBS-YOLOv5 structure, b-2: SPC network and b-3: SPPF$\_$1 structure.

In the default Neck section (shown in Fig. 4a), a combination of FPN+PAN ideas was used. The feature maps were fused with feature information extracted from Backbone’s C3, C4 and SPPF layers, respectively. Firstly fusion of high level features with the underlying features through the FPN network. It aimed to simultaneously exploit the high resolution of the underlying features and the rich semantic information of the high-level features to improve the detection of small objects. The different levels of feature information were then fused by the PAN structure. The purpose was to retain a more overly shallow location feature to further improve network performance. The final results after three inspection heads as pre-detection were 80 $\times$ 80, 40 $\times$ 40 and 20 $\times$ 20. We proposed the spatial pyramidal convolution module as the detection head for very small targets in the default Neck section (see Fig. 4b). It contained a 3 $\times$ 3 convolution, SPPF module, SPPF$\_$1 module and C3 module. The SPPF$\_$1 contained a 1 $\times$ 1 convolution and three parallel standard convolutions (with convolution kernel sizes of 3 $\times$ 3, 5 $\times$ 5 and 7 $\times$ 7). The Spatial Pyramid Convolution module took the output of Backbone’s P2 layer as input to the module.

Initially after SPPF block and SPPF$\_$1 block, the number of channels of the feature map was changed. In the next stage, the feature map was stitched together by the Concat operation. An output of the spatial pyramidal convolution module was used as a 160 $\times$ 160 detection head. Finally, we embed a recursive gated convolution on top of the default Neck.

### Efficient space-time interaction module

**Figure 5:**
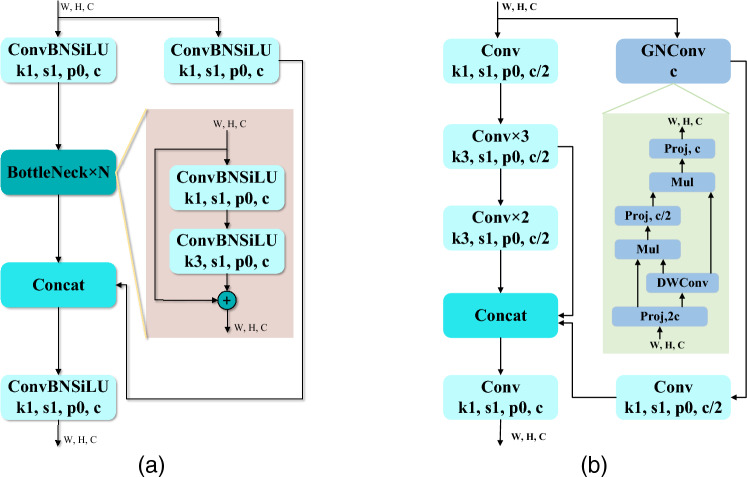
Efficient space-time interaction module (**a**) C3 block, (**b**) ESI block.

The default C3 module consisted mainly of 1 $\times$ 1 convolution and N $\times$ bottleneck modules. This module was the main module for learning about residual features. Its structure was divided into two branches (as shown in Fig. 5a). The left branch used an N $\times$ Bottleneck stack and 3 standard convolutional layers. The right-hand branch undergoed only one basic convolution module. Finally, the two branches were subjected to a concat operation. Despite its effectiveness in increasing the depth of the network, the C3 module was still inadequate in extracting feature information for small targets. This led us to propose the Efficient Spatio-Temporal Interaction module. The module was used to deepen the feature extraction network while at the same time extracting feature information about the target. The structure was divided into two main left and right branches (shown in Fig. 5b). First, the left branch consisted of N recursive gated convolution and 1 $\times$ 1 convolution layers. Its function was to achieve input adaptation and higher order spatial interaction through recursive gated convolution. Immediately afterwards, the feature map was fed into the 1 $\times$ 1 convolution module to change the feature dimension. Immediately afterwards, the feature map was fed into the 1 $\times$ 1 convolution module to change the feature dimension. The right-hand branch was a stack of standard convolutions and serves to extract a larger range of features and deeper features. Finally, the output of the two branches was subjected to a Concat operation.

In this case, the recursive gated convolution is implemented as follows.Supposed that $x\in {{R}^{HW\times C}}$ is the input feature. The projection features ${{p}_{0}}$ and $\left\{ {{q}_{k}} \right\} ,k\in \left[ 0,n-1 \right]$ are obtained by means of a linear projection function $\phi$. Its effect is to slice and dice the input features according to the number of channels, as shown in Eq. (4).

4 $$\begin{aligned} \left[ p_{0}^{HW\times {{C}_{0}}},q_{0}^{HW\times {{C}_{0}}},\cdots ,q_{n-1}^{HW\times {{C}_{n-1}}} \right] ={{\phi }_{in}}(x) , {{\phi }_{in}}(x)\in {{R}^{HW\times ({{C}_{0}}+\sum \nolimits _{0\le k\le n-1}{{{C}_{k})}}}} \end{aligned}$$

The sliced features are fed sequentially into the gated convolution for recursive operations as in Eq. (5), where $k=0,1,\ldots ,n-1$.

5 $$\begin{aligned} {{p}_{k+1}}={{f}_{k}}\left( {{q}_{k}} \right) \odot {{{g}_{k}}({{p}_{k}})}/{\alpha }\; \end{aligned}$$

Equation (2) scales the output features of each operation by ${1}/{\alpha }\;$. In the process of spatial interaction it is necessary to ensure that both are of the same dimension. Therefore, ${{g}_{k}}$ is the dimensional mapping function during the operation. Finally, the output *y* is obtained by recursive gated convolution, as in Eq. (6).

6 $$\begin{aligned} y=\phi ({{p}_{k+1}})\in {{R}^{HW\times C}} \end{aligned}$$

### Shallow bottleneck

**Figure 6:**
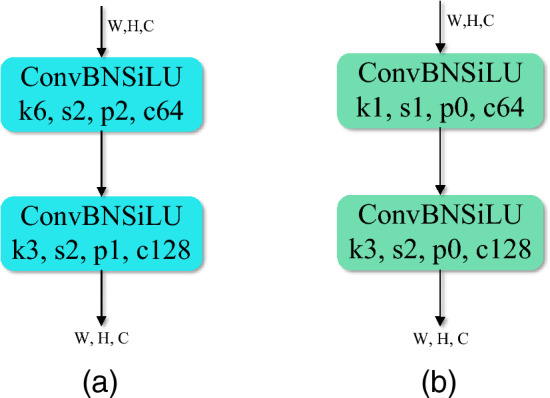
The default structure compared with the improved structure (**a**) default input, (**b**) shallow bottleneck input.

In the default YOLOv5 model, a 640 $\times$ 640 size image with 3 channels was given 2$\times$ downsampling through a 6 $\times$ 6 size convolution kernel with a step size of two. Convolution kernels with a length and width of 6 were not friendly to the retention of small target information. After 2$\times$ downsamplings, a great deal of detailed information about small targets was lost. Therefore, in our proposed GBS-YOLOv5 model, a large convolutional kernel was transformed into 1 $\times$ 1 and 3 $\times$ 3 convolutional kernels. The feature map was subsequently 2$\times$ downsampled after a 3 $\times$ 3, step 2 convolution kernel. The specific structure was shown in Fig. 6, where the default model was shown in the Fig. 6a and the improved model was shown in the Fig. 6b.

## Experimental results

All Experiments Conducted on VisDrone 2019 Dataset. For the fairness of the ablation experiments, we also tested excellent algorithms such as GBS-YOLOv5 and YOLOv3 based on the VisDrone dataset. The experimental results indicated that our proposed GBS-YOLOv5 had a good performance in terms of detection accuracy.

### Experimental setting

#### Experimental equipment

We used Windows 11 with a 12th Gen Intel(R) Core(TM) i5-12400 processor, 8 GB of RAM and an NVIDIA Geforce GTX3060 graphics card with 12 GB of Graphics memory.

#### Dataset

**Figure 7:**
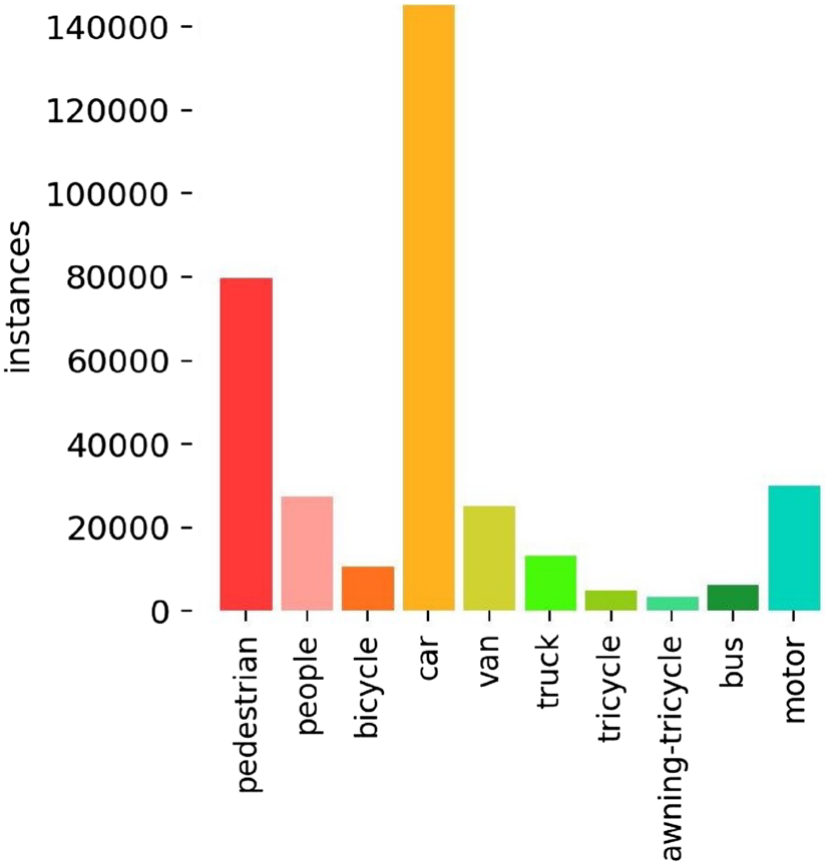
Volume information for each category in the VisDrone 2019 dataset.

Drones being loaded with cameras were already being used rapidly in agriculture, transport, aerial photography and other areas. As a result, the requirements for drones to collect visual data had become more stringent. To be persuasive, we used the VisDrone open source dataset for our experiments. The VisDrone dataset was collected by the AISKYEYE team at the Machine Learning and Data Mining Laboratory of Tianjin University. The datasets analysed during the current study were available in the VISDRONE repository (https://github.com/VisDrone/VisDrone-Dataset). It consisted of 26,908 frames and 10,209 images. This was captured by a drone camera and covers a wide area. These included location (14 different cities from China separated by thousands of kilometres), environment (urban and rural), objects (pedestrians, vehicles, bicycles, etc.) and density (sparse and crowded scenes). In addition to this, the datasets were collected using different drone platforms^18^ (i.e. different models of drones) in different scenarios and under different weather and lighting conditions^19^. The dataset was predefined with 10 category (pedestrian, person, car, van, bus, truck, motor, bicycle, awning-tricycle, and tricycle) bounding boxes. For the experiments, we used 10,209 still images and divided the dataset into a training set, a validation set, and a test set according to the official predefined. The dataset contained small targets, with heavy target occlusion and a large number of detection targets. In this case, the label information of the VisDrone dataset and the number of categories were shown in Fig. 7.

### Experimental detailed information

During the experiment, the AISKYEYE team has divided the dataset into a training set (6471 images), a validation set (548 images) and a test set (1610 images). In the experiment, we used the training parameters to set the training weights without official weights, the number of training epochs to 150, the batch size as 16, and the input size of 640 $\times$ 640. The training hyperparameters were set as follows. The initial learning rate was 0.01, where the stochastic gradient descent (SGD) method was 1E−2 and the stochastic optimiser Adam was 1E−3. The loss function in training was shown in equation 7. And to prevent overfitting phenomenon in training, we set the following hyperparameters for the characteristics of Visdrone dataset. Mosaic data enhancement was 1.0, scaling factor was 0.5, inversion factor was 0.1, mage HSV-value augmentation was 0.4, image HSV-saturation augmentation was 0.7, and image HSV-hue augmentation was 0.015, etc.

7 $$\begin{aligned} L\text {oss}=aLos{{s}_{obj}}+bLos{{s}_{box}}+cLos{{s}_{_{cls}}} \end{aligned}$$

In this case, the *Loss* in training contained three main aspects of loss: rectangular box loss ($L\text {os}{{\text {s}}_{box}}$), confidence loss ($L\text {os}{{\text {s}}_{obj}}$), and classification loss ($L\text {os}{{\text {s}}_{}}$). Loss weights *a*, *b*, *c* were set to: 1.0, 0.05, 0.5, respectively. Before starting training, the YOLOv5 model first calculated the best recall of the anchored boxes for this dataset. When the best recall was greater than or equal to 0.98, used the model default anchor box. Conversely, if the optimal recall did not reach the specified threshold, the model recalculated the appropriate anchor frame using the kmeans clustering algorithm. Through extensive experimentation, we had finalised the four inspection head anchor frame sizes for the GBS-YOLOv5 model, as shown in Table 1.

**Table 1:** Anchor frame size for the four inspection heads of the GBS-YOLOv5 model.

Detection head	Anchor frame size
Tiny detection head	[3, 4], [4, 9], [8, 6]
Small detection head	[7, 13], [13, 9], [12, 17]
Medium detection head	[23, 13], [19, 23], [41, 21]
Large detection head	[27, 44], [59, 40], [80, 86]

The official YOLOv5s model contained a total of five sub-models: YOLOv5l, YOLOv5m, YOLOv5n, YOLOv5s, YOLOv5x. These five types of models were diferent only in the parameters of the width and depth of the network. Due to the consideration of real-time algorithm, we chose YOLOv5s as the experimental control group, with network depth and width parameters of 0.33 and 0.50, respectively. In the training process, the training batch was adjusted to 16 and the works to 10. Image enhancement techniques such as mosaic and rotation were used. Uses a default model preheat round of 3 epochs.

### Experimental results

**Figure 8:**
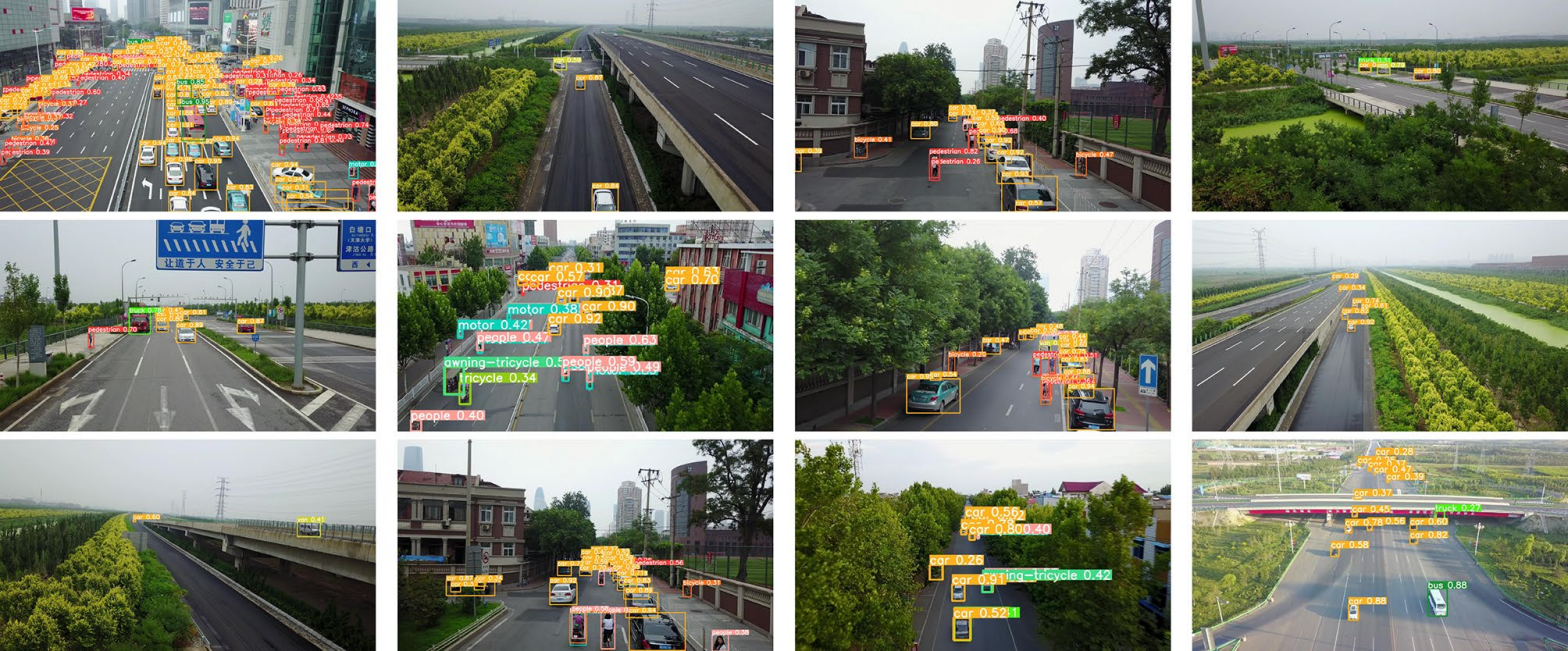
Partial detection results graph of GBS-YOLOv5 algorithm on Visdrone dataset.

We commenced by training YOLOv5 and GBS-YOLOv5 on the VisDrone 2019 dataset. A stochastic gradient descent method was used to optimize the model during the training process. We adjusted the training batch to 16, the momentum to 0.937 and the image input size to 640 $\times$ 640. The learning rate was set to 0.01. In addition, we used data enhancement methods such as mosaics and flips to extend the dataset during training. Of these, the experimental results for YOLOV5 and GBS-YOLOv5 were shown in Table 2. And Some GBS-YOLOv5 of the detection results were shown in Fig. 8.

**Table 2:** Compare with YOLOv5 and GBS-YOLOv5.

Models	P ($\%$)	R ($\%$)	mAP@0.5 ($\%$)	mAP@0.5:0.95 ($\%$)
YOLOv5s	41.4	32.4	31.3	16.5
**GBS-YOLOv5**	**43.2 (+ 1.8)**	**35.5 (+ 3.1)**	**35.3 (+ 4.0)**	**20.0 (+ 3.5)**

Significant values are in [bold].

Table 2, we clear noticed that our proposed algorithm has been improved by 1.8 $\%$ for precision (P), 3.1 $\%$ for recall (R), 4.0 $\%$ for mAP@0.5 and 3.5 $\%$ for mAP@0.5:0.95. Performance was further improved compared to the default YOLOv5s.

In addition to this, we compared the experimental results of the various algorithms on VisDrone. In order to compare the performance of GBS-YOLOv5 with various other algorithms more intuitively, we used mAP@0.5 as performance metrics. And we tuned the model scaling factors of YOLOv3 as well as yolov3-spp to ensure that the model scaling was the same as YOLOv5s. The network model has a depth multiple of 0.33 and a network width multiple of 0.50. The experimental results for each type of model were shown in Table 3.

**Table 3:** Comparison of the various algorithms on VisDrone2019 dataset.

	YOLOv3	YOLOv3-spp	YOLOv3-tiny	YOLOv5m	YOLOv5s	GBS-YOLOv5
Size	640 $\times$ 640	640 $\times$ 640	640 $\times$ 640	640 $\times$ 640	640 $\times$ 640	640 $\times$ 640
Pedestrian	41.2	41.3	18.9	42.7	39.2	42.3
People	33.2	34.1	17.7	34.0	31.3	32.8
Bicycle	9.16	9.8	3.4	12.0	9.6	8.8
Car	73.3	73.1	49.4	74.7	72.1	78.2
Van	31.5	31.6	11.2	36.8	32.9	39.0
Truck	23.2	23.7	9.6	30.2	26.3	30.9
Tricycle	17.3	17.2	8.4	19.2	15.9	22.2
Awning-tricycle	9.1	10.2	3.1	11.4	11.0	11.7
Bus	36.7	36.2	13.5	47.2	37.8	47.7
Motor	38.4	38.1	17.6	40.8	36.9	39.1
All (mAP@0.5)	31.3	31.5	15.3	34.9	31.3	35.3

Significant values are in [bold].

In Table 3, it could be easily seen that the proposed GBS-YOLOv5 model achieves better results in terms of accuracy compared to other detection models. The GBS-YOLOv5 had a 0.4$\%$ higher UAV detection accuracy than the YOLOv5m. It also outperformed YOLOv3, YOLOv3-spp and YOLOv3-tiny by 4.0$\%$, 3.8$\%$ and 20.0$\%$, respectively.

### Ablation experiment

In this section, the results of the ablation experiments were also presented and analysed. The ablation experiments were performed on the VisDrone 2019 dataset. We contemplated that the main contribution of this paper was based on the Backbone, Neck section. Therefore, the ablation experiment was divided into three parts. The results of the experiments were shown in Table 4.

**Table 4:** Comparison of the various algorithms on VisDrone2019 dataset.

Models	YOLOv5	YOLOv5+ESI (ours)	YOLOv5+SB (ours)	YOLOv5+SPC (ours)	GBS-YOLOv5 (ours)
ESI block	–	$\checkmark$	–	–	$\checkmark$
SB block	–	–	$\checkmark$	–	$\checkmark$
SPC block	–	–	–	$\checkmark$	$\checkmark$
size	640 $\times$ 640	640 $\times$ 640	640 $\times$ 640	640 $\times$ 640	640 $\times$ 640
Pedestrian	39.2	40.7	39.2	38.6	**42.3**
People	31.3	32.3	31.2	29.6	**32.8**
Bicycle	**9.6**	9.0	9.1	7.1	8.8
Car	72.1	73.8	71.3	76.3	**78.2**
Van	32.9	35.8	30.5	38.9	**39.0**
Truck	26.3	26.7	24.3	29.9	**30.9**
Tricycle	15.9	18.2	15.4	20.7	**22.2**
Awning-tricycle	11.0	10.3	10.8	11.0	**11.7**
Bus	37.8	41.1	40.1	46.3	**47.7**
Motor	36.9	37.7	37.5	37.3	**39.1**
P (all)	41.4	**43.2**	41.9	41.7	**43.2**
R (all)	32.4	34.0	31.9	33.2	**35.5**
All (mAP@0.5)	31.3	32.6	31.0	33.6	**35.3**

As seen in Table 4, YOLOv5+ESI, YOLOv5+SB, and YOLOv5+SPC were each added to YOLOv5 with the ESI block, SB block and SPC block, respectively. This would give us a better idea of whether the improvements we proposed were effective. On top of the default YOLOv5 model, only the ESI module was plused. The experimental results indicated that the accuracy of the model after the addition of ESI improved by 1.3$\%$ over the original model. In the same way, we added only the SB module and it could be seen that the accuracy was reduced by 0.3$\%$ compared to the original model. Since the SB module was designed to work against the SPC module, the 0.3$\%$ reduction in accuracy was negligible. We added the SPC module to the original model, and by comparing the experimental results, the addition of the SPC module improved the accuracy by 2.3$\%$ over the original model. Finally, the modules were assembled to form the GBS-YOLOv5. The precision of the ten categories was clearly seen in Table 4. The bicycle detection accuracy was reduced by 0. 8$\%$ compared to the original model. The detection accuracy for the remaining nine categories was superior to the original model.

## Conclusion

In this paper, the GBS-YOLOv5 algorithm based on YOLOv5 has been proposed. Three major contributions are listed. The first contribution is that an efficient spatiotemporal interaction module has been proposed to enhance the network’s ability to extract information about target features. The second contribution is that a shallow Bottleneck was proposed for the small target of UAV photography. It can enrich shallow feature information and help preserve small target information. The third point, we propose an spatial pyramid convolution module to improve small target detection performance through shallow information mining and feature fusion. The experiments demonstrated that the detection of our proposed GBS-YOLOv5 under the UAV dataset was better than that of other network models.

## Data Availability

All the images and experimental test images in this paper were from the open source VisDrone dataset. The VisDrone dataset was collected by the AISKYEYE team at the Machine Learning and Data Mining Laboratory of Tianjin University. The datasets analysed during the current study were available in the VISDRONE repository (https://github.com/VisDrone/VisDrone-Dataset).
